# Travelling by Rail

**Published:** 1869-12

**Authors:** 


### TRAVELLING BY RAIL.

A SECULAR paper says: "Want of good air, especially in Winter, is the greatest nuisance of railroad travelling in this country, and kills more persons than all the accidents of which we read such shocking accounts." It is not likely that any life has ever been lost by any such cause, and it is well enough for railroad travellers to think for themselves a moment, and possibly save a life thereby.

Of a dozen men and women, on a cold Winter's day, entering a car so crowded as to be barely able to find a seat, every window and door closed, and a fire in the stove, and no change made in door or window, not one will receive a serious injury, no, not one in a thousand, even if the ride is extended for hours.

But of an equal number entering another car, under the same circumstances, only that each will open a door or window nearest, one-half will be sick next day, if not nine out of ten. In any company of a dozen people, several may be found who will confess to having taken cold, or originated some unpleasant symptom, from riding in a car, or other vehicle, while in motion, with a near window open, giving rise to other symptoms of days' and weeks' and even years' duration. We venture the assertion, that not one person in ten will justly trace an attack of present sickness to riding in a close car with windows down. Such a thing may occasion some transient ill-feeling, as nausea, headache, and even fainting; but these are of momentary duration, usually passing away within half an hour—while any reader of ordinary reflection must know that many persons have been sent to their graves by sitting at or near the open window of a vehicle in motion. The difference between foul air and fresh air, as far as public carriages are concerned, is simply this: foul air does its worst by a transient fainting fit; fresh air excites coughs, colds, neuralgias, pleurisies, pneumonias, consumption, and death. No man or woman has any right, on entering a car or carriage, to open any window or door, without the express permission of every other person in it; and even with that, he is a selfish fool, inasmuch as, for his own comfort, he asks others to risk their's, their health, and their life. We are at all times safe, and largely benefitted, by being exposed to a still, cool atmosphere; and as certainly endanger life, by exposure to draughts of air.

